# Evaluation of Canal Transportation, Centring Ability, Shaping Volume and Debris Extrusion in Double-Curved Root Canals Prepared with Four Reciprocating Systems: An in vitro study

**DOI:** 10.4317/jced.64050

**Published:** 2026-04-25

**Authors:** Hernan Dario Muñoz-Alvear, Javier Caviedes-Bucheli, Hugo Roberto Muñoz, David Alvear, Juan Restrepo-Puetaman, Esteban Arciniegas-Guzman, Juan Diaz-Villacis, Luis Lopez-Moncayo, Néstor Ríos-Osorio

**Affiliations:** 1 Postgraduate Endodontics Department, School of Dentistry, Universidad Cooperativa de Colombia, Pasto, Colombia; 2 Centro de Investigaciones Odontológicas, Pontificia Universidad Javeriana, Bogotá, Colombia; 3 Endodontics Department, Universidad de San Carlos, Guatemala City, Guatemala; 4 Computer, Electrical and Mathematical Sciences and Engineering (CEMSE), King Abdullah University of Science and Technology (KAUST), Thuwal, Saudi Arabia; 5 Endodontic Research Department, CES University, School of Dentistry, Medellin, Colombia; 6 Faculty of Dentistry, Institución Universitaria Visión de las Américas, Medellín, Colombia

## Abstract

**Background:**

This in vitro study aims to evaluate canal transportation, centring ability, volumetric change, and apical debris extrusion of four reciprocating systems using CBCT, machine-learning segmentation, and the Myers-Montgomery method.

**Material and Methods:**

Forty-eight 3D-printed maxillary canine resin teeth with standardised double-curved canals were randomly allocated to Reciproc Blue (RB), WaveOne Gold (WOG), One Reci (OR), or RC-One (n = 12). All canals were prepared at body temperature using manufacturer-recommended kinematics. Pre- and post-instrumentation CBCT scans (voxel size 70 µm) were segmented using supervised clustering to obtain canal geometry. Transportation and centring ability were analysed at 0.5-10 mm from the apex. Canal volume change was calculated from three-dimensional reconstructions. Apically extruded debris was collected and weighed. Non-parametric statistics (Kruskal-Wallis with Dunn's post hoc test) were applied ( = 0.05).

**Results:**

RC-One exhibited the lowest debris extrusion (median 0.032 mg), whereas WOG produced the greatest extrusion. RC-One also showed the lowest canal transportation (median 0.27), significantly lower than the other systems (p &lt; 0.001), while WOG demonstrated the highest transportation (mean 0.80). RC-One achieved the highest centring ability (mean 0.69), whereas WOG showed the lowest (mean 0.42). The smallest volumetric enlargement was observed with RC-One (99.57%), significantly lower than WOG and RB (p &lt; 0.01). WOG produced the greatest volume increase (137.23%).

**Conclusions:**

RC-One demonstrated the most favourable overall performance, with superior centring ability, minimal transportation, conservative shaping, and the lowest apical debris extrusion. These outcomes may be related to the interaction between its alloy properties, geometric design, and reciprocating kinematics. In contrast, WOG showed greater canal deviation and debris extrusion under the present experimental conditions.

## Introduction

Shaping curved root canals is technically demanding. About 80-85% of canals exhibit some degree of curvature, increasing the risk of canal transportation, ledging, perforation, or over-instrumentation-reported in 5-44% of cases depending on curvature complexity and operator skill. Such errors alter apical anatomy, reduce cleaning efficacy, and jeopardise treatment outcomes (1 , 2). Recent advances in nickel-titanium (NiTi) instrumentation, including heat-treated alloys and refined reciprocating kinematics, have enhanced flexibility, cyclic fatigue resistance, and centring ability in complex canal anatomies (3). These innovations aim to minimize procedural errors and promote a conservative, biologically respectful shaping approach that preserves the canal's original curvature and spatial orientation (4). When optimally balanced, shaping achieves a selective and conservative dentine removal, providing adequate enlargement for irrigation and disinfection while preventing structural weakening or excessive apical enlargement (5). While reciprocating systems enhance shaping efficiency, centring ability, and preservation of canal anatomy, these mechanical dynamics can also generate and push dentinal debris towards the apex, leading to neurogenic inflammation of the periodontal ligament due to neuropeptide release. The severity of this response correlates with the amount of extruded debris, leading to post-endodontic pain in up to 65% of cases (6). Thus, the physical design and kinematics that optimize shaping performance also govern the biological impact of instrumentation, making debris extrusion a crucial parameter in evaluating the overall safety of reciprocating systems (7). For this study, four reciprocating instruments were selected: One Reci (Micro-Mega, Coltene Group, Besançon, France), Plex RC-One (Jining Orodeka Medical Equipment Co., Ltd., Jining, Shandong, China), Reciproc Blue (VDW GmbH, Munich, Germany), and Wave One Gold (Dentsply Maillefer, Ballaigues, Switzerland). Canal transportation, centring ability, and shaping volume can be reliably assessed using computed tomography coupled with software-based three-dimensional reconstruction. This technique provides a non-destructive and clinically relevant method that allows precise evaluation of internal canal geometry while preserving specimen integrity (8). Building on the analytical precision of computed tomography, recent advances in computational engineering have further enhanced data interpretation in endodontic research through the integration of artificial intelligence (AI). In this context, machine-learning approaches-particularly clustering algorithms-facilitate the automated categorization of imaging datasets into groups or "clusters" based on shared radiodensity patterns and spatial characteristics (9). This process enhances image contrast and definition, allowing the accurate discrimination of dentine, voids, and prepared canal spaces during three-dimensional reconstruction. By segmenting voxels with similar intensity values, clustering not only refines the geometric assessment of canal shaping but also provides a more objective and reproducible analysis compared with traditional observer-dependent methods. Additionally, the Myers and Montgomery method was employed to enable precise and reproducible quantification of debris extruded through the apical foramen (10). Despite advances in reciprocating single file systems and AI-assisted 3D evaluation, comparative data on their performance in double curved canals remain limited. Accurate assessment of shaping efficiency, centring ability, dentine removal, and debris extrusion is essential, as deviations can compromise treatment outcomes and increase post-operative discomfort (11). Investigating these systems in controlled invitro conditions thus provides crucial insights for optimizing safe and biologically conservative root canal preparation. In view of the foregoing, this study employed CBCT with machine learning segmentation and 3D reconstruction, combined with the Myers and Montgomery methodology aiming to evaluate canal transportation, centring ability, shaping volume, and apical debris extrusion of four reciprocating nickel-titanium systems-Reciproc Blue (RB), Wave One Gold (WOG), One Reci (OR), and RC-One-after biomechanical preparation of double-curved resin canals.

## Material and Methods

**Figure 1:**
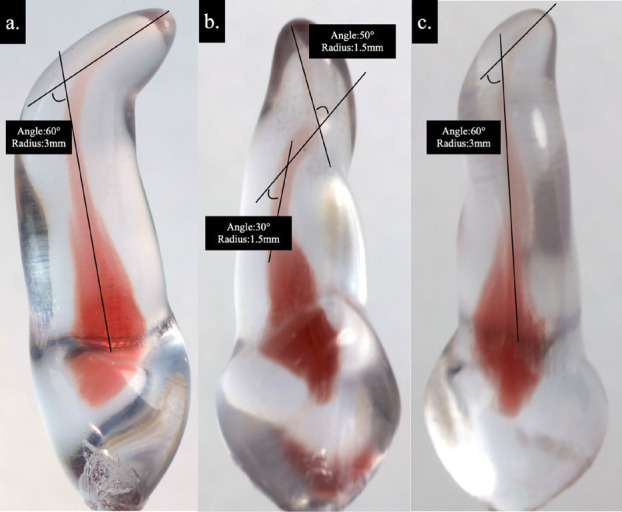
Canal curvature configurations of the 3D-printed resin teeth. (A) Mesial view: angle 60°, radius 3 mm. (B) Palatal view showing an S-shaped canal with a coronal curvature of 50° (radius 1.5 mm) and an apical curvature of 30° (radius 1.5 mm). (C) Buccal view: angle 60°, radius 3 mm.

- Sample Selection and Size Calculation The sample size was calculated using analysis of variance (ANOVA) in G*Power 3.1.9 for Mac (Heinrich Heine University, Düsseldorf, Germany), based on data from a previous study with a comparable design (12). Assuming an effect size of 0.5, a power of 0.80, = 0.05, and four experimental groups, 12 samples per group were required. Consequently, 48 three-dimensionally printed resin teeth (Jining Orodeka Medical Equipment Co., Ltd., Jining, Shandong, China) replicating a maxillary right canine with double curvature were used. Canal curvature was standardized according to Schneider's method (13) (Fig. 1).

1

**Figure 2:**
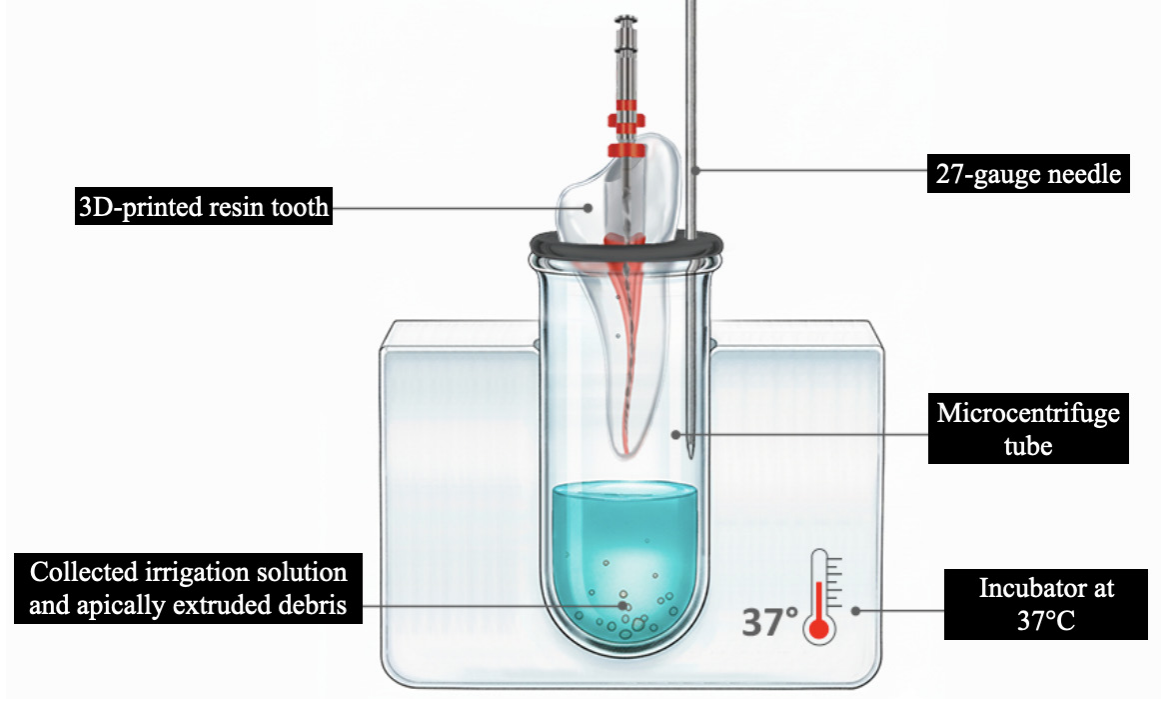
Experimental setup used for the collection of apically extruded debris. Each 3D-printed resin tooth was positioned inside a microcentrifuge tube to collect the extruded material. A 27-gauge needle was inserted alongside the tooth to equalize internal and external air pressure during instrumentation. The entire assembly was maintained at 37 °C, and the apically extruded debris and irrigant were collected in the tube and subsequently weighed.

Access cavities were prepared using a round bur No. 2 Diatech (Coltene/Whaledent AG, Altstätten, Switzerland) in a high-speed handpiece, and canal patency was confirmed with a No. 10 K-file (Dentsply Sirona, Ballaigues, Switzerland) under a dental operating microscope (Zumax Medical Co., Ltd., Suzhou, China). - Experimental Design Specimens were randomly assigned to four groups (n = 12): Group 1 - OR (170° CCW/60° CW, 400 rpm); Group 2 - RB (150° CCW/30° CW, 300 rpm); Group 3 - RC-One (160° CCW/40° CW, 500 rpm); and Group 4 - WOG (150° CCW/30° CW, 350 rpm). Canal preparation was performed according to each manufacturer's protocol, standardizing motion, torque, and speed parameters. - Canal Preparation, Shaping, and Measurement of Extruded Debris Each resin tooth was secured at the opening of an Eppendorf tube using silicone, and a 27-gauge needle was inserted to equalise internal pressure. The assemblies were incubated at 36-37°C for 15 minutes (Dry Bath Heater, Thermo Scientific, Waltham, MA, USA) to simulate intraoral conditions. Canal preparation was performed by a single operator using an E-connect S+ motor (Eighteeth Medical Technology Co., Ltd., Changzhou, China) under continuous irrigation with 9 mL of distilled water per tooth. A new file was used for each specimen, and instrumentation was terminated upon reaching working length. Apically extruded debris was collected and weighed according to the Myers and Montgomery method using an analytical balance (Adam PW-254, Adam Equipment, UK; precision = 10-4 g) (Fig. 2).

2

**Figure 3:**
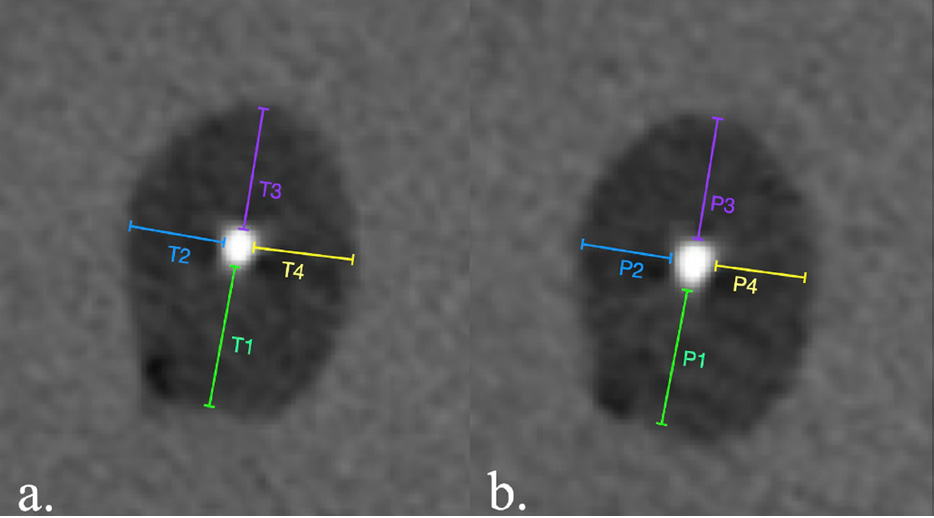
a–b. Axial CBCT slices showing the initial measurements (T1–T4) and the final measurements (P1–P4), obtained before and after instrumentation using the same reference axes.

- Pre- and Post-Instrumentation CBCT Scans and Contrast Medium Application A rectangular resin base with 12 compartments (2.5 × 2.5 cm each) was 3D printed to stabilize the samples using condensation silicone (Speedex Trial Kit, Coltene, Switzerland). The teeth were positioned and labelled from D0 to D11, maintaining a consistent spatial orientation. All groups were scanned using a CBCT system (Carestream CS 8200 3D, Carestream Dental LLC, Atlanta, GA, USA) at the highest available resolution (90 kV, 4 mA, 15 s exposure time). Both pre- and post-instrumentation scans were performed using a CBCT system (Carestream CS 8200 3D, Carestream Dental LLC, Atlanta, GA, USA) at the highest available resolution (90 kV, 4 mA, 15 s exposure) and with an iodinated contrast agent (Omnipaque®, Iohexol 300 mg/mL; GE Healthcare, Chicago, IL, USA) to enhance canal visualization and accurately delineate the internal anatomy. - Clustering, Software Design, and Image Visualization Image processing was implemented in Python to process the pre- and post-instrumentation CBCT scans, without altering native data. Slice thickness and pixel spacing were verified and standardized (0.15 mm = 1 pixel). The region of interest was limited from the root apex to the cusps to minimize background noise. Supervised clustering, optimised using the elbow method, was employed for accurate segmentation of radiopaque structures. The cluster representing dental and canal anatomy was isolated, denoised using morphological operations, and reconstructed to calculate three-dimensional volumes and bounding boxes (D0-D11) for each specimen. - Visualization Software A dedicated visualization and measurement software was developed in Python version 3.13.7 to analyze the segmented images. The program enabled calibration of a zero-reference point and the placement of measurement markers along each canal, allowing precise quantification of the parameters described below. Additionally, the software allowed verification of tooth diameter and export of all measurement data in CSV format for statistical analysis. - Analysis and Measurement of Models Determination of Canal Transportation and Centring ability Canal transportation was evaluated in both the mesiodistal (M-D) and buccopalatal (B-P) directions by comparing pre- and post-instrumentation CBCT images. The initial measurements (T) represented the dentine thickness before instrumentation, while the post-instrumentation measurements (P) corresponded to the dentine thickness after shaping. The following distances were assessed: T1 - from the palatal root surface to the uninstrumented canal wall; T2 - from the mesial root surface to the uninstrumented canal wall; T3 - from the buccal root surface to the uninstrumented canal wall; and T4 - from the distal root surface to the uninstrumented canal wall. The same measurements were repeated after instrumentation and designated as P1-P4, respectively (Fig. 3 a-b).

3

**Figure 4:**
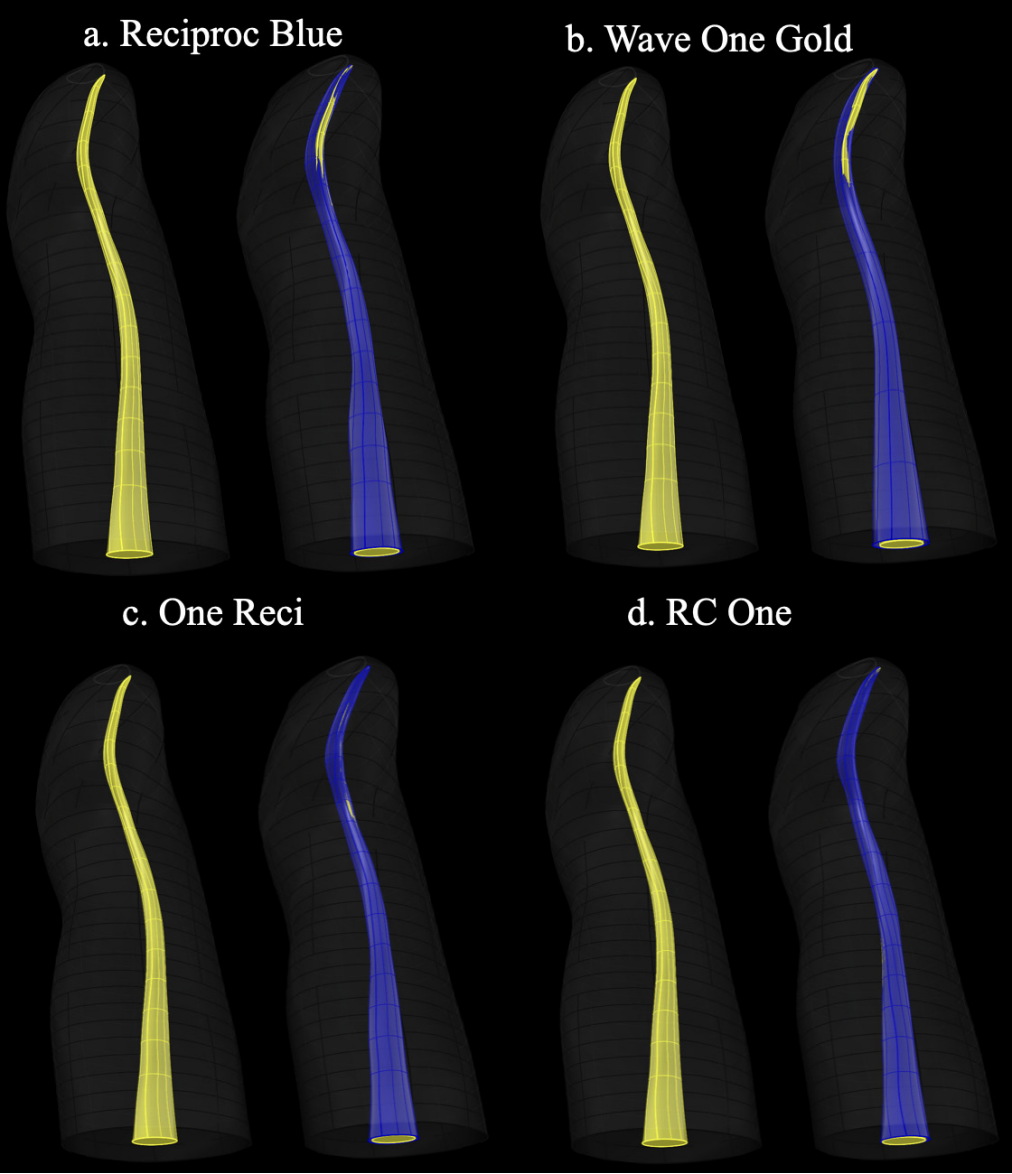
3D palatal-view reconstructions of the root canal show the original anatomy (yellow) and the prepared canal (blue) for each reciprocating system (a) Reciproc Blue, (b) WaveOne Gold, (c) One Reci, and (d) RC-One. The overlays reveal marked differences in anatomical fidelity: some instruments deviate from the native trajectory, particularly in curvature zones.

Measurements were recorded at 0.5 mm and at 1-10 mm along the canal length. Canal centring and transportation were evaluated following a previously published protocol, where a centring ratio of 1 indicates perfect alignment with the canal axis, and values approaching 0 reflect diminished centring ability. Conversely, a transportation value of 0 indicates no deviation; positive values denote mesial or palatal displacement, while negative values indicate distal or buccal deviation (4 , 12). - 3D Reconstruction Process Forty-eight (48) CBCT scans were obtained before and after root canal preparation. Axial cross-sections were generated at 0.5 mm intervals up to 10 mm for each specimen. The canal contours were delineated using the polyline command and exported sequentially to Rhinoceros software (Robert McNeel &amp; Associates, Washington, USA). Each slice was colour-coded and aligned at 0.5 mm intervals to reconstruct the canal geometry in three dimensions. Pre- and post-instrumentation models were superimposed to assess morphological variations. Canal volumes were automatically calculated using the software's Volume function (Fig. 4).

4

- Statistical analysis The statistical analysis was performed using SPSS Statistics 21.0 (Armonk, New York: IBM Corp.). Apical debris extrusion was expressed as the weight (mg) of collected debris. Data normality was assessed using the Shapiro-Wilk test. Median values with 95% confidence intervals (CI) were reported. Intergroup comparisons were performed using the Kruskal-Wallis test, followed by Dunn's post hoc test. Canal volume increase was expressed as the percentage change between final and initial canal volumes. Normality was verified with the Shapiro-Wilk test. Median values with 95% CIs were reported, and intergroup differences were analysed using the Kruskal-Wallis and Dunn's post hoc tests. Canal transportation was calculated using the transportation formula, where 0 indicates no deviation and increasing values reflect greater displacement. Canal centring was assessed using the centring ratio, ranging from 0 (least centred) to 1 (perfectly centred). Data normality for both parameters was evaluated using the Kolmogorov-Smirnov test. Mean values and 95% confidence intervals (CIs) were reported at 0.5, 1-10 mm from the apex. Intergroup comparisons were performed using the Kruskal-Wallis test followed by Dunn's post hoc pairwise analysis to determine statistically significant differences.

## Results

- Apical Debris Extrusion The Shapiro-Wilk test indicated that the data were not normally distributed (p &lt; 0.001). The highest amount of debris extrusion was observed in the WOG group, followed by RB, OR, and RC-One, respectively. The Kruskal-Wallis test revealed statistically significant differences among groups (p = 0.014). Dunn's post hoc pairwise comparisons showed significant differences between RB and RC-One (p = 0.0204) and between WOG and RC-One (p = 0.0020) (Table 1).

Table 1

- Canal Volume increase The Shapiro-Wilk test indicated that the data were not normally distributed (p = 0.041). The greatest percentage of canal volume increase was observed in the WOG group, followed by RB, OR, and RC-One, respectively. The Kruskal-Wallis test revealed statistically significant differences among groups (p &lt; 0.001). Dunn's post hoc pairwise comparisons showed significant differences between WOG and OR (p = 0.0099), WOG and RC-One (p = 0.0001), and RB and RC-One (p = 0.0037) (Table 2).

Table 2

- Canal Transportation The Kolmogorov-Smirnov test indicated that the data were not normally distributed (p &lt; 0.001). The highest transportation values along the entire canal were observed in the WOG group, followed by RB, OR, and RC-One, respectively. The Kruskal-Wallis test revealed statistically significant differences among groups (p &lt; 0.001). Dunn's post hoc pairwise comparisons showed significant differences for all group comparisons except between RB and OR (p = 0.555) (Table 3).

Table 3

Table 4 summarises the transportation values recorded at each canal section analysed, from 0.5 mm up to 10 mm.

Table 4

- Canal Centring Ability The Kolmogorov-Smirnov test indicated that the data were not normally distributed (p &lt; 0.001). The highest centring ability along the entire canal was observed in the RC-One group, followed by OR, RB, and WOG, respectively. The Kruskal-Wallis test revealed statistically significant differences among groups (p &lt; 0.001). Dunn's post hoc pairwise comparisons showed significant differences among the systems, except between Reciproc Blue and One Reci (p = 0.2540) (Table 3). Table 4 summarises the centring values recorded at each canal section analysed, from 0.5 mm up to 10 mm.

## Discussion

Reciprocating single-file systems are designed to optimize shaping in complex anatomies by preserving canal trajectory and apical foramen while minimizing debris extrusion. Their kinematics reduce the screw-in effect and cyclic fatigue, lowering dentinal stress and allowing controlled dentine removal without excessive volumetric change. This is especially relevant in double-curved canals, as approximately 84% of root canals exhibit curvature in at least one plane, and 17.5% present a secondary curvature. When these curvatures exceed 25°, they are classified as severe, posing a significant endodontic challenge (13 , 14). This study employed 3D-printed acrylic teeth with artificial canals (Fig. 1), providing standardized, homogeneous samples that enabled balanced experimental groups under comparable conditions without ethical constraints. These models also enabled high-resolution CBCT imaging with 70 µm slices, allowing precise visualization and measurement of canal anatomy. However, the results should be interpreted with caution when extrapolating to clinical practice (5). The systems evaluated in this study were single-file reciprocating instruments - OR, RB, RC-One, and WOG - which represent the most extensively studied reciprocating systems, demonstrating superior fatigue resistance due to advanced designs and innovative alloys (7). All instruments had a #25 tip size and a taper ranging from 6% to 8%, according to manufacturer specifications (15). Root canal preparation followed each manufacturer's instructions to minimize operator-related bias. The use of standardized 3D-printed teeth ensured consistent comparisons under controlled conditions. Preparation was performed at body temperature (36-37 °C), as the thermo-dependent behaviour of NiTi alloys affects their martensitic-austenitic transition, increasing stiffness and potentially influencing shaping performance, transportation, and centring ability (16). Initial assessment and canal preparation were evaluated using CBCT scans with 70-µm slices, providing adequate resolution to detect morphological changes. This allowed qualitative and quantitative three-dimensional analysis referenced to calibrated zero points for precise measurement of all variables (17). A radiopaque contrast medium containing nanoscale iodine particles was employed to enhance visualization and ensure accurate assessment of narrow canal spaces (18). Each tooth was segmented using AI-based clustering to group pixels with similar contrast for pre- and post-instrumentation analysis of canal transportation and centring. Three-dimensional reconstruction in Rhinoceros software provided measurements with approximately 1% error (5). Apical debris extrusion was quantified following the Myers and Montgomery method, a controlled and reproducible gold-standard approach (10). The results of the present in vitro study indicate that the RC-One system exhibited the lowest canal transportation, highest centring ability, minimal volumetric change, and least debris extrusion. This trend was consistently observed when compared with WOG, OR and RB. These outcomes may be associated with specific instrument characteristics, including tip design, transition angle, cross-sectional geometry, helical angle, taper, alloy composition, and kinematic motion. RC-One and OR feature an inactive tip combined with a short transition angle, allowing controlled cutting and smooth advancement along the root canal while minimising transportation and apical debris extrusion (7). RB likewise, features an inactive tip; however, its long transition angle increases apical pressure, which accounts for its greater propensity for canal deformation (19). WOG, in contrast, has a semi-active tip that enhances apical penetration; however, its long transition angle widens the contact area, reducing cutting efficiency and consequently increasing apical debris extrusion and canal transportation by compromising tip stability (6). The RC-One file exhibits a constant 6% taper and a slim modified convex triangular cross-section with three cutting points, providing strong apical stability and generous coronal space for efficient debris removal. This geometry then transitions to two S-shaped cutting points, enhancing anatomical adaptation and minimising apical debris extrusion (0.032 mg) (7). The OR file exhibits a constant 6% taper and a thicker convex triangular cross-section within the apical 3 mm, a configuration that produces greater apical debris extrusion compared with RC-One. Beyond this region, its geometry transitions into an S-shaped cross-section from 6 mm to 16 mm (0.053 mg) (20). The RB file has an 8% taper in its apical 3 mm, followed by a decreasing taper of 4% between 4 and 5 mm, and then stabilising at 5%. Its S-shaped cross-section with two cutting edges results in reduced centring ability and increased apical extrusion, most likely related to the regressive taper configuration of the instrument (0.062 mg) (21). The parallelogram cross-section of the WOG file, with one or two active cutting edges, increases the instrument's mass and mechanical instability. When combined with its variable taper-7% in the apical 3 mm-this geometry contributes to greater apical debris extrusion (0.074 mg) (22). RC-One and OR feature a constant 6% taper, optimising shaping while preserving anatomy and limiting dentine removal and lateral forces (volume increase: RC 103%, OR 115%). RB has an 8% apical taper over the first 3 mm that reduces to 4% coronally, concentrating stress apically and producing transportation with a 115% volume increase. WOG presents a 7% apical taper with a wide core, resulting in aggressive cutting, poor curvature adaptation, a 133% volume increase and the greatest debris extrusion (7 , 21). RC-One, OR, and RB systems exhibit a variable helical angle that enhances adaptability and shaping efficiency. Conversely, WOG's constant helical angle limits canal conformity, increases deformation and apical pressure, saturates debris evacuation zones, and accounts for its greater debris extrusion (19). The RC-One system retains martensitic flexibility at 37 °C, allowing optimal canal adaptation, uniform shaping, and controlled debris extrusion (7). The OR system is temperature-sensitive and may shift to austenite above 40.3 °C, reducing canal conformity and increasing volume change and debris extrusion. The RB system (Blue Wire, Af 38 °C) becomes rigid at body temperature, promoting aggressive apical cutting and greater extrusion due to its tip design and transition angle (19). The WOG system (Gold Wire, Af &gt; 46 °C) may locally transform to austenite from frictional heat, decreasing flexibility and inducing lateral cutting, maximal canal deformation, and apical debris extrusion (22 , 23). The RC-One system (160°/40°, 500 rpm) provides centred and stable cutting dynamics with minimal debris extrusion. OR (170°/60°, 350 rpm) demonstrates reduced mechanical stability due to its alloy transition behaviour and asymmetric reciprocation angles. RB (150°/30°, 300 rpm) exhibits unstable shaping performance attributable to its regressive taper and S-shaped cross-sectional design. WOG (150°/30°, 300 rpm), owing to its parallelogram cross-sectional profile and regressive taper, generates torque peaks and increased vibrational load, thereby compromising canal preservation (7 , 24). When comparing the results of this study with previously published findings, WOG showed the highest canal transportation and the lowest centring ability at 2-9 mm, a pattern associated with its large core, parallelogram cross-section, 7% apical taper, semi-active tip, and limited cutting-zone stability (7). RB produced less transportation than WOG in both S-shaped and natural canals, whereas One Shape (Coltene/Micro-Mega, Besançon, France) demonstrated superior centring performance owing to its triangular three-point cross-section-findings that align with the results of the present study (25). Reports on debris extrusion are inconsistent: some found higher values for RB than WOG in double-curved canals, others reported no difference when glide paths were standardised (12 , 26). In ex vivo models, OR extruded more debris than WOG but less than RB (27). No data exist for RC-One; however, its CM alloy, as in Plex V 2.0, has been associated with lower debris extrusion (12). No prior studies have assessed canal volume increase using comparable 3D methods. The main limitation of this study is the use of standardized acrylic resin replicas rather than extracted human teeth. While these models permit strict experimental control, their physical and mechanical properties differ from natural dentine, particularly in terms of hardness, thermal behaviour during instrumentation, and debris characteristics. These differences may affect the interaction between the instrument and canal walls, potentially influencing outcomes such as canal transportation, centring ability, volumetric changes, and debris extrusion. Therefore, caution is warranted when extrapolating these findings to clinical practice. Nevertheless, artificial models offer significant methodological advantages for comparative endodontic research. Standardized replicas allow precise control of anatomical variables, including canal curvature, radius, diameter, and taper, thereby minimizing the inherent variability of natural root canals. This level of standardization enables direct comparison between instrumentation systems under identical anatomical conditions, which is often challenging with extracted teeth. Advances in resin-based and three-dimensional printed models have further improved dimensional accuracy and reproducibility, allowing complex canal anatomies to be consistently replicated across experimental groups. Thus, despite this limitation, the present model remains a valid and widely accepted approach for evaluating the shaping performance of endodontic instruments under controlled and reproducible conditions. Accordingly, the results should be interpreted within this experimental context when considering their clinical implications (28 - 30).

## Conclusions

Within the limitations of this in vitro study, RC-One exhibited the most favourable performance among the evaluated reciprocating single-file systems, demonstrating superior centring ability, lower canal transportation, minimal volumetric change, and reduced apical debris extrusion. These outcomes likely reflect the combined effects of its geometric design, reciprocating kinematics, and CM alloy composition. In contrast, Wave One Gold showed greater canal transportation, increased volumetric change, and higher debris extrusion under the same experimental conditions. Therefore, given the methodological limitations, these results should be interpreted with caution when extrapolating to clinical practice.

## Figures and Tables

**Table 1:** Apical debris extrusion in mg after canal preparation with different reciprocating files.

Reciprocating File	N	Median	95% Confidence Interval	Significant Post-hoc (Dunn)
Reciproc Blue	12	0.062	0.0468 – 0.127	vs RC-One (p = 0.0204)
Wave One Gold	12	0.074	0.0644 – 0.126	vs RC-One (p = 0.0020)
One Reci	12	0.053	0.0331 – 0.101	-
RC-One	12	0.032	0.0228 – 0.0432	-

The Kruskal–Wallis test showed significant differences among the four systems (p = 0.014). Dunn’s post-hoc analysis revealed that RC-One produced significantly less apical debris extrusion compared with Reciproc Blue (p = 0.0204) and WaveOne Gold (p = 0.0020). No other pairwise comparisons reached statistical significance.

**Table 2:** Percentage of canal volume increase after canal preparation with different reciprocating files.

Reciprocating File	N	Median	95% Confidence Interval	Significant Post-hoc (Dunn)
Reciproc Blue	12	127.97	115.73 – 134.97	vs RC-One (p = 0.0037)
Wave One Gold	12	137.23	126.03 – 140.81	vs One Reci (p = 0.0099)vs RC-One (p = 0.0001)
One Reci	12	112.90	104.95 – 124.89	-
RC-One	12	99.57	96.06 – 109.15	-

The Kruskal–Wallis test revealed significant differences in the percentage of canal volume increase among the systems (p < 0.001). Dunn’s post-hoc analysis showed that WaveOne Gold produced the greatest enlargement, with significantly higher values than One Reci (p = 0.0099) and RC-One (p = 0.0001). Reciproc Blue also produced significantly greater volume increase than RC-One (p = 0.0037). No significant differences were observed between Reciproc Blue and WaveOne Gold, or between Reciproc Blue and One Reci.

**Table 3:** Transportation and centring ability along the entire canal after preparation with different reciprocating files.

Reciprocating File	Transportation			Centring Ability			
	Mean	95% Confidence Interval	Significant Post-hoc (Dunn)	Mean	95% Confidence Interval	Significant Post-hoc (Dunn)	
Reciproc Blue	0.49	0.442 – 0.538	vs WOG (p < 0.001) vs One Reci (p = 0.5550).	0.47	0.457 – 0.523	vs WOG (p < 0.001) vs One Reci (p = 0.2540)	
Wave One Gold	0.80	0.724 – 0.876	-	0.42	0.389 – 0.451	-	
One Reci	0.48	0.438 – 0.522	vs WOG (p < 0.001)	0.53	0.479 – 0.541	vs WOG (p < 0.001)	
RC-One	0.27	0.243 – 0.297	vs Reciproc Blue (p < 0.001) vs Wave One Gold (p < 0.001),vs One Reci (p < 0.001).	0.69	0.646 – 0.694	vs Reciproc Blue (p < 0.001) vs Wave One Gold (p < 0.001),vs One Reci (p < 0.001).	



Significant differences were found in the mean canal transportation among the four systems (Kruskal–Wallis, p < 0.001). Post-hoc Dunn testing demonstrated that RC-One produced the lowest transportation, being significantly more conservative than Reciproc Blue (p < 0.001), WaveOne Gold (p < 0.001), and One Reci (p < 0.001). No significant difference was observed between Reciproc Blue and One Reci (p = 0.5550). Similarly significant differences were found in canal centring ability among the systems (Kruskal–Wallis, p < 0.001). RC-One achieved the greatest centring ability, being significantly more centered than Reciproc Blue, Wave One Gold, and One Reci (all p < 0.001). Wave One Gold showed the lowest centring ability, with significantly lower values compared with Reciproc Blue (p < 0.001) and One Reci (p < 0.001). No significant difference was found between Reciproc Blue and One Reci (p = 0.2540).

**Table 4:** Canal transportation and centring ability at different distances from the apex after preparation with four reciprocating file systems.

Transportation at each mm of the canal after preparation with different reciprocating files											
Reciprocating File	0.5	1	2	3	4	5	6	7	8	9	10
Reciproc Blue	0.48	0.55	0.58	0.44	0.46	0.48	0.52	0.49	0.46	0.48	0.41
Wave One Gold	0.72	0.57	0.89	0.75	0.85	0.83	0.78	1.02	0.85	0.85	0.72
One Reci	0.37	0.48	0.56	0.45	0.53	0.46	0.54	0.48	0.61	0.48	0.34
RC-One	0.24	0.26	0.32	0.15	0.31	0.23	0.23	0.36	0.29	0.26	0.36
p (Kruskal-Wallis)	<0.01	<0.01	<0.01	<0.01	<0.01	<0.01	<0.01	<0.01	<0.01	<0.01	0.34
Centring ability at each mm of the canal after preparation with different reciprocating files											
Reciprocating File	0.5	1	2	3	4	5	6	7	8	9	10
Reciproc Blue	0.49	0.54	0.41	0.54	0.43	0.52	0.40	0.38	0.49	0.57	0.59
Wave One Gold	0.42	0.49	0.34	0.47	0.42	0.49	0.39	0.37	0.43	0.39	0.46
One Reci	0.58	0.50	0.46	0.46	0.44	0.62	0.47	0.51	0.39	0.58	0.65
RC-One	0.74	0.71	0.67	0.76	0.66	0.72	0.68	0.61	0.57	0.67	0.55
p (Kruskal-Wallis)	<0.01	<0.01	<0.01	<0.01	<0.01	<0.01	<0.01	<0.01	0.13	0.01	0.25

Values represent the mean canal transportation and centring ability measured at 0.5–10 mm from the apex after instrumentation with Reciproc Blue, WaveOne Gold, One Reci, and RC-One. Transportation values closer to 0 indicate less deviation from the original canal path, whereas centring ratios closer to 1 indicate better canal centring ability. Intergroup comparisons at each level were performed using the Kruskal–Wallis test (α = 0.05).

## Data Availability

The datasets used and/or analyzed during the current study are available from the corresponding author.
